# Optimal target localization for botulinum toxin A in treating splenius muscles dystonia based on the distribution of intramuscular nerves and spindles

**DOI:** 10.1007/s12565-025-00831-8

**Published:** 2025-03-03

**Authors:** Xiaojiao He, Sifeng Wen, Xuan Liu, Yutong Li, Shengbo Yang

**Affiliations:** 1 Department of Radiology, The Affiliated Hospital of Zunyi Medical University, Zunyi, 563003 People’s Republic of China; 2 Department of Basic Medicine, Xinpu New Developing Area, Zunyi Medical University, 6 West University Road, Zunyi, 563099 People’s Republic of China; 3 Department of Clinical Medicine, Xinpu New Developing Area, Zunyi Medical University, 6 West University Road, Zunyi, 563099 People’s Republic of China; 4 Department of Anatomy, Xinpu New Developing Area, Zunyi Medical University, 6 West University Road, Zunyi, 563099 People’s Republic of China

**Keywords:** Botulinum toxin A, Intramuscular nerves, Muscle spindle abundance, Splenius muscles, Target localization

## Abstract

Botulinum toxin A injection is commonly used to treat splenius muscle dystonia; however, the optimal injection site within the muscle remains unidentified. This study identified the optimal target for botulinum toxin A based on the distribution of intramuscular nerves and spindles in the splenius muscles. This study included 24 adult individuals. The curve connecting the external occipital protuberance and the third thoracic spinous process was the longitudinal reference line (line L). The curve connecting the external occipital protuberance and the mastoid process was the horizontal reference line (line H). Modified Sihler's staining showed an intramuscular nerve-dense region in the splenius muscles. Muscle spindle abundance was calculated after hematoxylin and eosin staining. The center of the region of the highest muscle spindle abundance was localized using computed tomography. The projection points (P and P') of the center of the region of the highest muscle spindle abundance behind and in front of the neck, position of P (P_L_ and P_H_) projected onto the L and H lines, and depth of the center of the region of the highest muscle spindle abundance were determined under the Syngo system. P_L_, P_H_, and depth of the center of the region of highest muscle spindle abundance of splenius capitis and splenius cervicis muscles were 17.33% and 40.59% of the L line, 42.42% and 60.44% of the H line, and 26.30% and 32.60% of the PP' line, respectively. These results will provide morphological guidance for improved efficiency and efficacy of target localization for botulinum toxin A treatment for splenius muscle dystonia.

## Introduction

The splenius muscles, comprising the splenius capitis and splenius cervicis muscles, are named after their V-shaped grips on the cervical spine. Unilateral contraction of the splenius muscles results in bending and turning of the head and neck to the same side, whereas their bilateral contraction results in the straightening of the head and neck (Blouin et al. 2007). The splenius muscles, which produce the peak extension of the neck, excessively elongate and become stiff from chronic injury due to prolonged desk work (Grob et al. 2007; Cheon et al. 2020). This condition can result in cervical pain or migraine, which affects over 30% of the population (Cohen 2015). Patients with central nervous system diseases, such as stroke, brain trauma, and spinal cord injury, may experience splenius spasms, restricted movement, and headaches (Phadke et al. 2019). In these cases, the splenius muscles are under high tension, and clinical management involves intramuscular botulinum toxin A (BTX-A) injection to reduce this tension (Supiot et al. 2018). BTX-A blocks the release of acetylcholine from the presynaptic membrane of the motor end plate, thereby inhibiting muscle excitation (Isner-Horobeti et al. 2016).

However, the position of the motor endplate band of the splenius muscles is currently unknown; therefore, the exact anatomical site of intramuscular BTX-A injection remains undetermined. Currently, staining of the motor endplate band is limited to fresh specimens. Previous studies suggest that the intramuscular nerve-dense region (INDR) is in the same location as that of the motor endplate band and can be used as an alternative target for BTX-A (Amirali et al. 2007). However, recent studies have suggested that muscle spindles are allometric stretch receptors crucial for regulating the alpha–gamma loop in muscle spasms (Phadke et al. 2013). Although the muscle spindle abundance in the INDR is high, its distribution is not uniform. BTX-A should be injected into areas with a high muscle spindle distribution to efficiently block the superimposed excitation effect of the α–γ loop and minimize the impact on the extrafusal muscle to improve the quality of rehabilitation (Phadke et al. 2013).

Previous research has demonstrated that in animals, injecting BTX-A into the center of the region of the highest muscle spindle abundance (CRHMSA) yields better results for treating muscle spasms compared to those achieved by injecting it at the traditional site at the thickest part of the muscle belly (Yu et al. 2023). Therefore, this study aimed to determine if the CRHMSA within the INDR of the splenius muscles is the optimal target for BTX-A for the treatment of abnormal increases in muscle tone in the splenius muscles and for improving the efficiency and efficacy of this treatment.

## Materials and methods

### Specimen and ethical approval

Anatomical specimens from 24 Chinese adults (12 male, 12 female; age: 35–75 [57.7 ± 11.5] years old) with no history of neuromuscular diseases or head and neck malformations were collected. The causes of death among these donors were cancer, heart disease, and accident. Among them, 12 (male, 6; female, 6) body donors were fixed with formalin for intramuscular nerve staining, and 12 (male, 6; female, 6) were frozen for hematoxylin and eosin (HE) staining and CRHMSA localization. Body donors were collected and used with the consent of the Ethics Committee of Zunyi Medical University (#2020–1-008). The date of the permission for our experiments is February 14, 2020.

### Gross anatomy observation, measurement, and reference line design

Twelve anatomical specimens placed in a prone position were fixed with formalin. A horizontal incision was made from the external occipital protuberance to the mastoid process, and a longitudinal incision was made from the external occipital protuberance to the seventh thoracic spinous process. Skin and subcutaneous fat were turned over from the medial to lateral side against the surface of the muscle. Trapezius, rhomboid, superior posterior serratus, and levator scapulae were turned outward by cutting off their origin and insertion. The insertion of the sternocleidomastoid muscle was cut and turned forward. The splenius capitis and splenius cervicis muscles were exposed to observe their morphology, muscle fiber arrangement, origin and insertion, adjacent relationship, the passage of extramuscular nerves, nerve entry point, and presence of accompanying vessels. To describe the superior and the inferior relationships, and the medial and lateral relationships between the block targets of the splenius muscles and the bony landmarks, we conceptualized a longitudinal reference line (L line) for the near-skin connection between the external occipital protuberance (point A) and the spinous process of the third thoracic vertebra (point B). A horizontal reference line (H line) was conceptualized for the near-skin connection between the external occipital protuberance and the mastoid process (point C).

### Modified Sihler’s intramuscular nerve staining

After gross anatomical observation, splenius capitis and cervicis muscles on both sides were removed, and fat and fascia on the muscle surface were removed. Subsequently, modified Sihler's staining was performed (Hu et al. 2024); pigmentation was removed by immersing the tissue in 3% potassium hydroxide and 0.2% hydrogen peroxide solution for 3–4 weeks; decalcification was conducted in Sihler's I solution for 4 weeks; samples were dyed in Sihler's II solution for 4 weeks, decolorized using Sihler's I solution for 3–24 h, and neutralized using 0.05% lithium carbonate solution for 1–2 h with stirring. Transparency was achieved using a 40–100% gradient glycerin for 1 week each. The distribution of the intramuscular nerve branches was observed using an X-ray viewing box, photographs were taken, and distribution pattern sketches were drawn. Vernier calipers were used to measure the percentage position of the INDR on muscle length and width.

### HE staining and muscle spindle abundance count

After thawing and dissecting 12 cryopreserved body donors, the splenius muscles were carefully exposed, and the corresponding position of INDR was removed, according to the results of Sihler's staining. The INDR of the splenius capitis muscle was divided into three equal parts: lateral, middle, and medial. The INDR of the splenius cervicis muscle was divided into three equal parts: upper, middle, and lower. The samples were weighed, fixed in formaldehyde, dehydrated, wax-embedded, and sectioned into continuous 5-μm slices. HE staining was performed, and the muscle spindles were observed and counted using an optical microscope. Using the formula (Spn = 20.5 mn^0.49^) (Banks 2006), where mn is the muscle weight, the predicted number of muscle spindles (Spn) was calculated. The actual number was divided by the predicted number to obtain the abundance of muscle spindles in the three INDRs. By comparison, the region with the highest spindle abundance and the positions of CRHMSA were determined.

### Spiral computed tomography (CT) localization of CRHMSA

Muscle blocks of the same size as the INDR were cut from other body areas to fill the gap during muscle spindle staining. CRHMSA was labeled with barium sulfate mixed with 801 Glue (Wenzhou Co., China) and sutured in situ layer by layer. Subsequently, it was subjected to spiral CT scanning and three-dimensional reconstruction. Under the Syngo system, the body surface projection point of the CRHMSA on the back of the neck was denoted as the P point (the puncture point perpendicular to the skin). The intersection points of the horizontal line passing through point P with the L line and the vertical line with the H line were respectively denoted as P_L_ and P_H_ (A-P_L_ = L', A-P_H_ = H'). The lengths of L and L' and H and H' were measured. The ratios L'/L × 100% and H'/H × 100% were calculated. The point that projected the P point onto the skin in front of the neck after passing through the CHRMSA point was designated as the P' point. The length of P-CRHMSA and PP' was measured, and P-CRHMSA/PP' × 100% was calculated to determine the self-percentile position of CRHMSA puncture depth.

### Statistical analysis

The experimental data were processed using SPSS 29.0 software (IBM Corporation, Armonk, NY, USA). Results are expressed as percentage (± S)%, eliminating the influence of individual differences in height and weight. Since the data were normally distributed, one-way ANOVA was used to compare muscle spindle abundance among different parts. A paired t-test was used for comparison between left and right sides, and an independent sample *t*-test was used for comparison between males and females. *P* < 0.05 was considered statistically significant.

## Results

### Gross anatomical findings

**Fig. 1:**
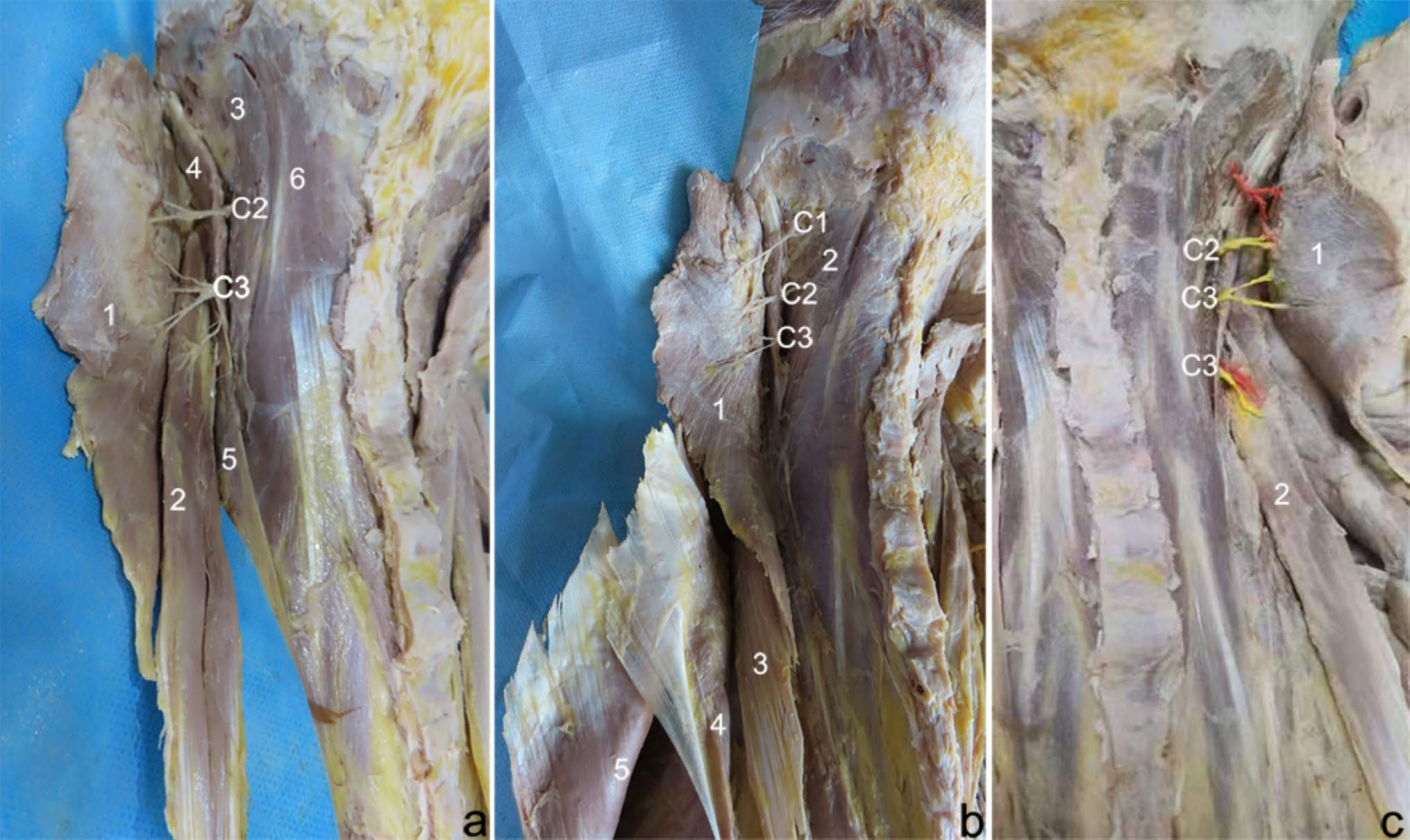
Gross anatomy of the splenius muscles innervation. **a** Common type of innervation of the splenius muscles (left side). 1 = splenius capitis muscle, 2 = splenius cervicis muscle, 3 = longissimus capitis muscle, 4 = longissimus cervicis muscle, 5 = iliocostalis cervicis muscle, 6 = semispinosus capitis muscle. C2/C3 = the nerves that innervate the splenius muscles. **b** Special type of splenius capitis innervation (left side). 1 = splenius capitis muscle, 2 = longissimus capitis muscle, 3 = splenius cervicis muscle, 4 = serratus posterior superior muscle, 5 = rhomboid muscle. C1/C2/C3 = the nerves that innervate the splenius muscles. **c** Innervation of splenius muscles and accompanying vessels (right side). 1 = splenius capitis muscle, 2 = splenius cervicis muscle, C2/C3 = nerve branches of the splenius muscles

The innervation of the splenius capitis muscle, 91.67% (44/48 sides), was derived from the posterior lateral branches of nerves C2 and C3. These nerve branches often pass through the space between the longissimus capitis and longissimus cervicis muscles and enter the deep surface of the lateral to middle–upper part of the splenius capitis muscle. The C2 nerve branches typically split into two before entering the muscle, and the C3 nerve branches are usually divided into two to four branches (Fig. 1a). Among the innervation of the splenius capitis muscle, 8.33% (4/48 sides) originated from the posterior lateral branches of the C1–C3 nerves. The nerve branches from C1 and C2 often passed through the longissimus capitis muscle before merging into the splenius capitis muscle (Fig. 1b). The nerves of the splenius cervicis muscle originated from the posterior lateral branches of the C3. The posterior lateral branches of C3 passed between the longissimus cervicis and iliocostalis cervicis to reach the middle–upper deep surfaces of the splenius cervicis muscle and were usually divided into two to three branches before entering the muscle (Fig. 1a). The C2 nerve branch of the splenius capitis muscle and the C3 nerve branch of the splenius cervicis muscle were often accompanied by blood vessels at the entry muscle (Fig. 1c).

### Intramuscular nerve distribution and muscle spindle abundance

**Fig. 2:**
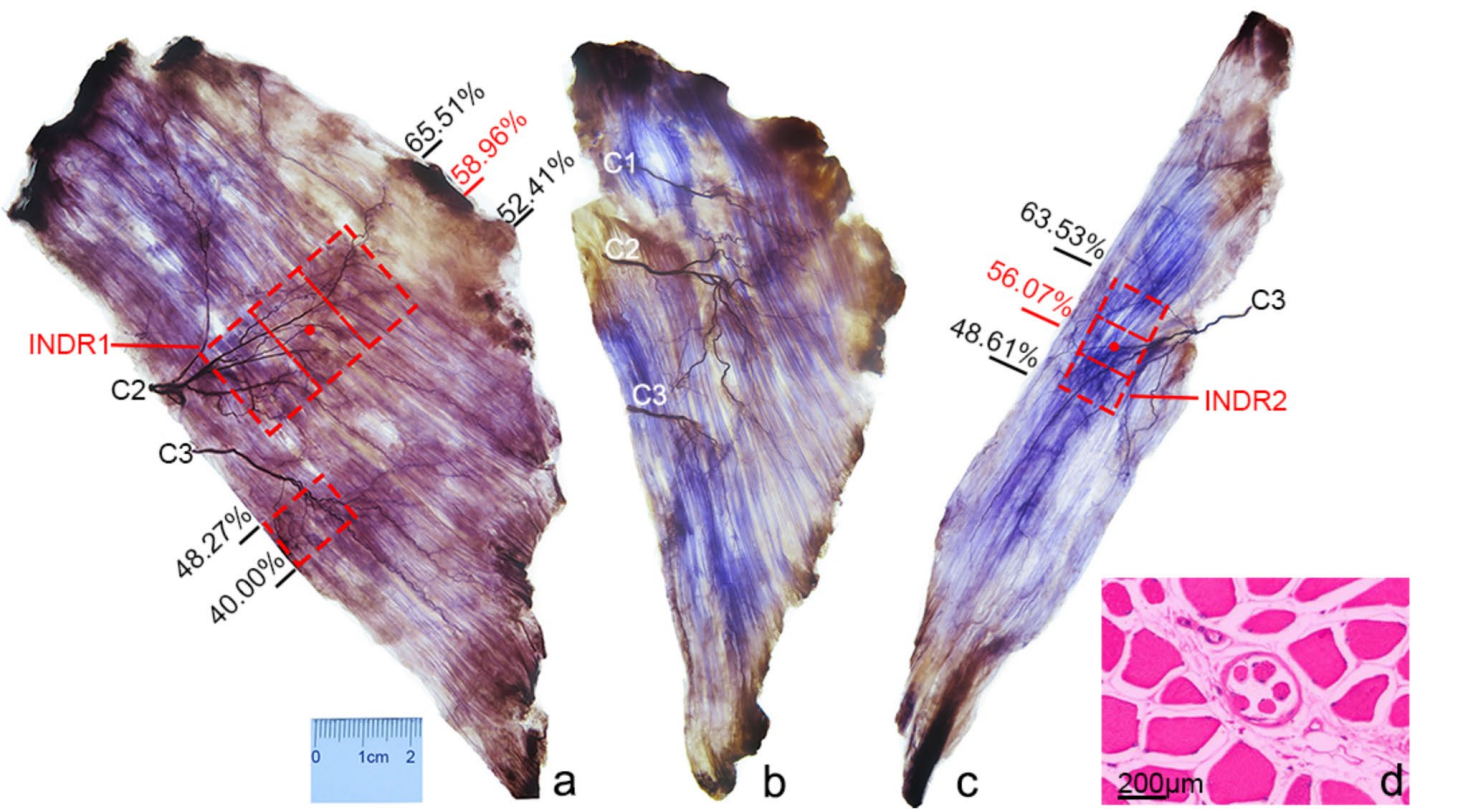
Sihler's staining of the splenius muscles and the CRHMSA. **a** Sihler's staining of the left splenius capitis muscle (common type, superficial view). The red box and dot indicate the INDR and CRHMSA, respectively. **b** Special type of Sihler's staining of the splenius capitis muscle. **c** Sihler's staining of the splenius cervicis muscle. **d** HE staining showing representative muscle spindle in splenius capitis muscle

**Fig. 3:**
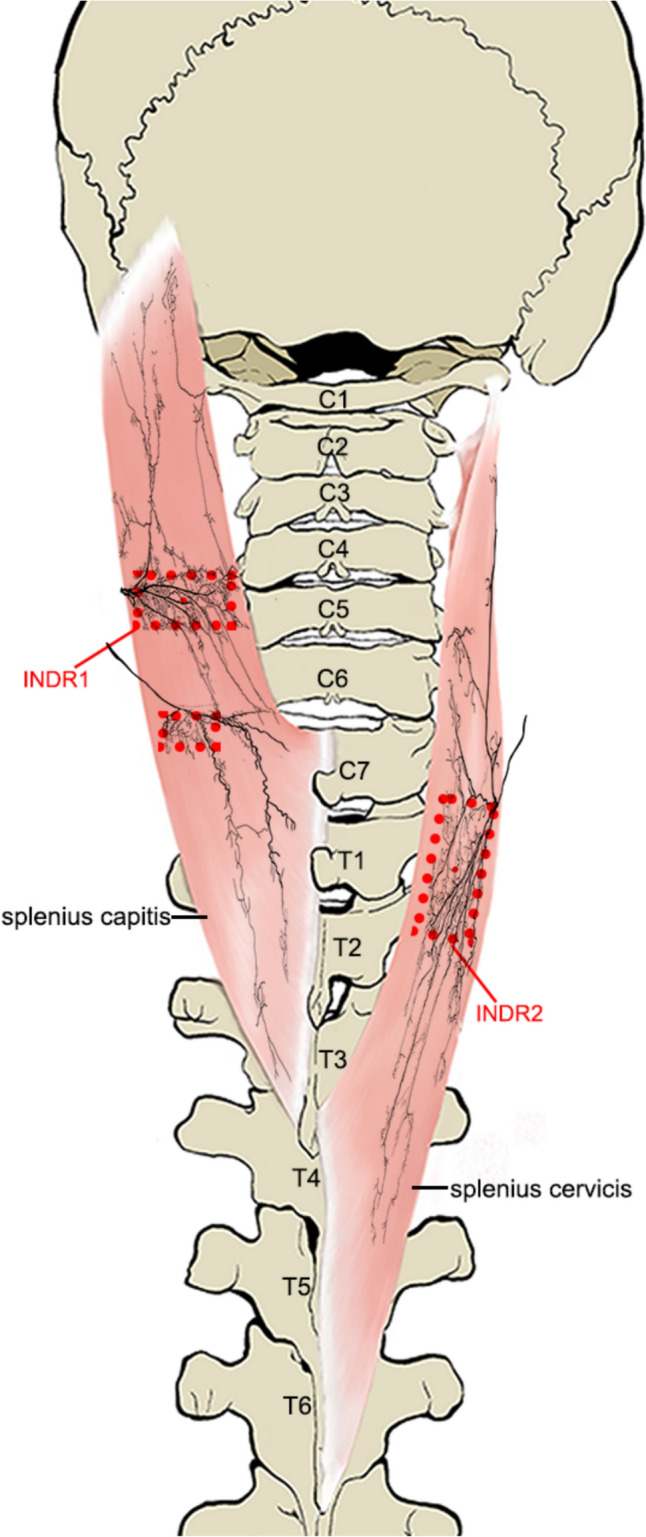
Sketch of intramuscular nerve distribution pattern in the splenius muscles. The left and right sides of the sketch, respectively, show the positions of the INDR of the splenius capitis and splenius cervicis muscles

Among the common types of nerve innervation in the splenius capitis muscle, after the C2 nerve branch of the splenius capitis entered the muscle, its upper primary branch ran obliquely superomedially, with relatively sparse branches. Its main trunk ran parallel to the medial side, with many arborized branches along the way; these arborized branches communicated with the branches of its lower primary branches. A rectangular intramuscular nerve-dense region (INDR1) with an area of (6.95 ± 0.76) cm^2^ was formed at 52.25 ± 0.77% to 62.80 ± 3.05% of the muscle belly length. The nerve branch of the splenius capitis from C3 entered the muscle and ran obliquely inferomedial. Although an INDR was formed at 31.52 ± 3.84% to 41.20 ± 3.41% of the muscle belly length, it was not the main nerve innervating the muscle because of the small nerve-dense region. The objects were not included in the target locations (Figs. 2a, 3). In the special type of nerve innervation of the splenius capitis muscle, the nerve fibers from C1 had the same innervation range as that of the upper primary branch of the C2 nerve branch in common nerve innervation types (Fig. 2b). After the splenius cervicis nerve branch from C3 entered the muscle, it ran obliquely superomedial, inferomedial, and inferolateral and emitted arborized branches that communicated with each other. An intramuscular nerve-dense region (INDR2) area of approximately (5.12 ± 1.39) cm^2^ was formed at 59.57 ± 1.83% to 63.96 ± 1.47% of the muscle belly length (Figs. 2c, 3). The abundance of muscle spindles in INDR1 and INDR2 was the highest in the middle parts, and their CRHMSA were located at 58.27 ± 0.51% and 56.18 ± 0.53% of the splenius capitis and splenius cervicis muscle lengths, respectively (Fig. 2a, c, d). Table 1 shows the muscle spindle abundance in each part within each INDR. Muscle spindle abundance among the different parts was compared, and the difference was statistically significant (*P* < 0.05). However, no significant difference was found in muscle spindle abundance between males and females or the left and right sides (*P* > 0.05) (Table 2).

**Table 1:** Comparison of muscle spindle abundance between different parts of INDR in splenius muscles (mean ± SD)

INDRs	Parts of INDR	Muscle weight (g)	Actual number of muscle spindles	Predicted number of muscle spindles	Relative muscle spindle abundance
INDR_1_	lateral	0.63 ± 0.08	30.25 ± 1.36	16.27 ± 1.03	1.86 ± 0.09*
	Middle	0.62 ± 0.08	33.00 ± 2.68	16.23 ± 1.08	2.04 ± 0.20
	medial	0.64 ± 0.09	29.71 ± 1.81	16.37 ± 1.19	1.81 ± 0.16*
INDR_2_	Upper	0.55 ± 0.07	21.04 ± 1.57	15.22 ± 1.05	1.39 ± 0.09※
	Middle	0.56 ± 0.09	24.17 ± 1.55	15.37 ± 1.18	1.58 ± 0.08
	Lower	0.55 ± 0.08	20.50 ± 2.00	15.28 ± 1.08	1.34 ± 0.09※

INDR_1_ and INDR_2_ represent the intramuscular nerve-dense region of the splenius capitis and splenius cervicis, respectively

^*^, ※, compared with the middle part of INDR1 and INDR2, respectively, *P* < 0.05

**Table 2:** Comparison of spindle abundance of each part of the INDR between males and females and between the left and right sides in splenius muscles (mean ± SD)

INDRs	Parts of INDR	Muscle spindle abundance							
		men (*n* = 6)	women (*n* = 6)	*t*	*P*	Left side (*n* = 12)	Right side (*n* = 12)	*t*	*P*
INDR_1_	lateral	1.83 ± 0.02	1.90 ± 0.03	−1.70	0.11	1.87 ± 0.11	1.86 ± 0.08	0.10	0.93
	Middle	1.97 ± 0.06	2.11 ± 0.04	−1.86	0.08	1.99 ± 0.23	2.08 ± 0.15	−1.44	0.18
	medial	1.79 ± 0.18	1.83 ± 0.14	−0.67	0.51	1.86 ± 0.20	1.76 ± 0.10	1.82	0.10
INDR_2_	Upper	1.34 ± 0.09	1.40 ± 0.09	−0.84	0.41	1.39 ± 0.08	1.38 ± 0.10	0.50	0.63
	Middle	1.57 ± 0.02	1.58 ± 0.02	−0.26	0.80	1.58 ± 0.09	1.57 ± 0.07	0.47	0.65
	Lower	1.36 ± 0.07	1.33 ± 0.11	0.77	0.45	1.35 ± 0.08	1.33 ± 0.10	0.27	0.79

### Spiral CT localization of CRHMSA

**Fig. 4:**
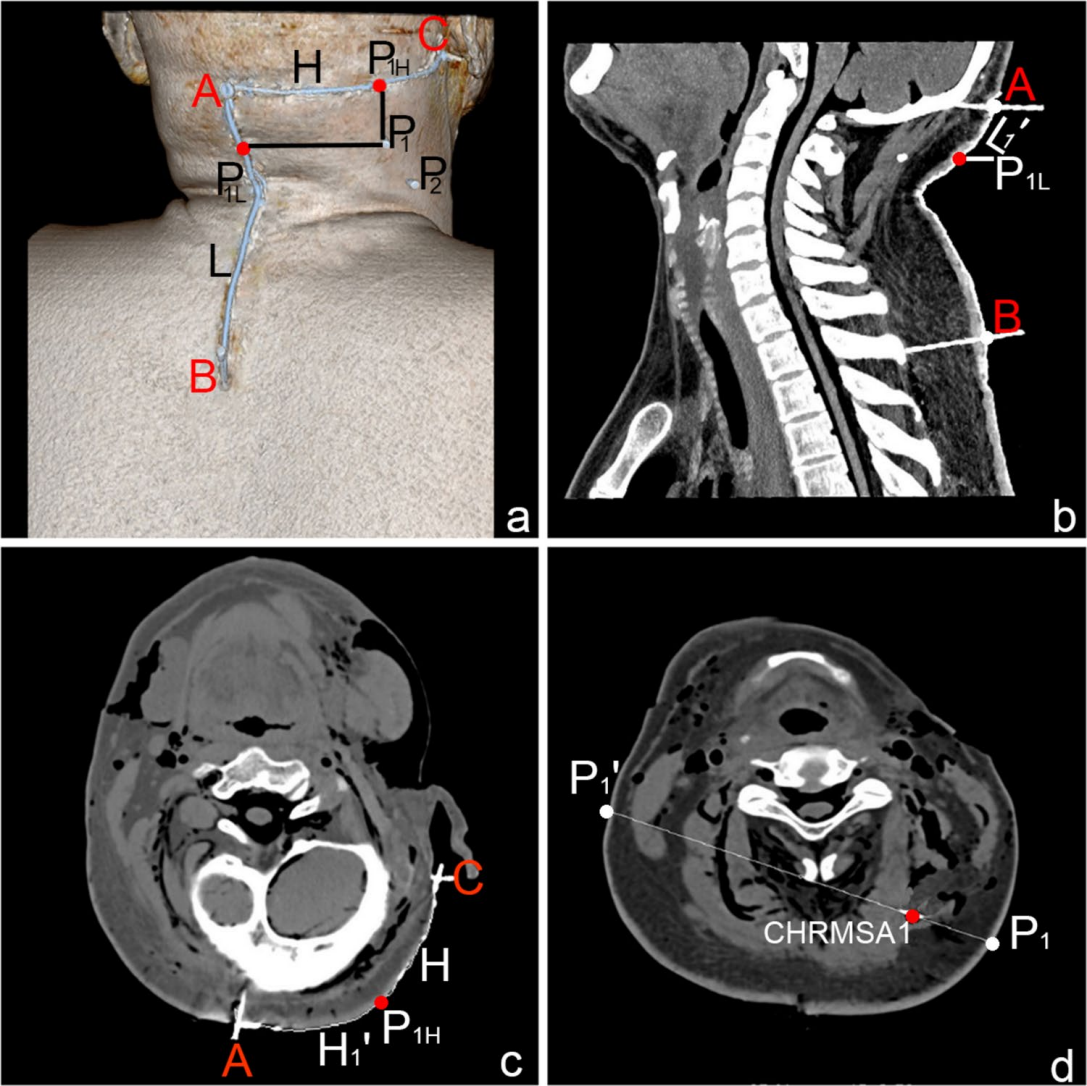
Spiral CT image of CRHMSA localization of the right splenius capitis muscle. **a** Body surface projection position of the CRHMSA and its reference line. A = external occipital protuberant, B = spinous process of the third thoracic vertebra, C = mastoid process, P_1_ = body surface projection point of the CRHMSA in the splenius capitis muscle, P_2_ = body surface projection point of the CRHMSA in the splenius cervicis muscle, AB = L, AC = H; A-P_1_L = L_1_', A-P_1_H = H_1_'. **b** Measurement of the length of the L and L_1_' lines on the sagittal section through line AB. **c** Measurement of the length of the H and H_1_' lines on the cross-section through line H. **d** Measurement of the depth of CRHMSA_1_ through point P_1_

The centers with the highest muscle spindle abundances in the splenius capitis and splenius cervicis muscles were designated as CRHMSA1 and CRHMSA2, respectively. Table 3 lists the percentage positions of P_L_ and P_H_ for each CRHMSA on the L and H lines and the depth of the CRHMSA. Comparisons of the data between the left and right sides and between males and females (Tables 4, 5) showed no statistical significance (*P* > 0.05). This study used the CRHMSA localization of the splenius capitis muscle as an example to illustrate the CT localization images (Fig. 4).

**Table 3:** P_L_ and P_H_ positions on the L and H lines and the depth of CRHMSA in splenius muscles (mean ± SD)

CRHMSAs	P_L_ on line L (L'/L%)	P_H_ on line H (H'/H%)	Depth of CRHMSA (P-CRHMSA/PP'%)
CRHMSA_1_	17.33 ± 2.13	42.42 ± 1.76	26.30 ± 3.43
CRHMSA_2_	40.59 ± 2.11	60.44 ± 2.45	32.60 ± 1.42

CRHMSA_1_ and CRHMSA_2_ represent the centers with the highest muscle spindle abundances of the splenius capitis and splenius cervicis muscles, respectively

**Table 4:** Comparison of the P_H_ and P_L_ positions on the H and L lines and the depth of CRHMSAs in splenius muscles between the left and right sides (mean ± SD)

CRHMSAs	P_L_ on line L (L'/L%)				P_H_ on line H (H'/H %)				Depth of CRHMSA (P-CRHMSA/PP'%)			
	Left side (*n* = 12)	Right side (*n* = 12)	*t*	*P*	Left side (*n* = 12)	Right side (*n* = 12)	*t*	*P*	Left side (*n* = 12)	Right side (*n* = 12)	*t*	*P*
CRHMSA_1_	17.15 ± 1.48	17.33 ± 2.13	0.39	0.70	42.71 ± 1.82	42.13 ± 1.65	1.29	0.22	26.16 ± 2.78	26.44 ± 3.97	0.16	0.87
CRHMSA_2_	40.57 ± 2.20	40.61 ± 2.02	0.17	0.86	60.40 ± 3.06	60.47 ± 1.62	0.07	0.94	32.64 ± 1.31	32.56 ± 1.52	0.29	0.77

**Table 5:** Comparison of the P_H_ and P_L_ positions on the H and L lines and the depth of CRHMSAs in splenius muscles between males and females (mean ± SD)

CRHMSAs	P_L_ on line L (L'/L%)				P_H_ on line H (H'/H %)				Depth of CRHMSA (P-CRHMSA/PP'%)			
	Males (*n* = 6)	Females (*n* = 6)	*t*	*P*	Males (*n* = 6)	Females (*n* = 6)	*t*	*P*	Males (*n* = 6)	Females (*n* = 6)	*t*	*P*
CRHMSA_1_	17.24 ± 1.48	17.25 ± 2.14	0.22	0.831	42.41 ± 1.76	42.52 ± 1.81	0.00	1.00	26.26 ± 3.45	26.35 ± 3.42	0.17	0.87
CRHMSA_2_	40.59 ± 2.41	40.58 ± 1.62	0.09	0.927	60.08 ± 1.92	60.58 ± 2.74	0.58	0.57	32.63 ± 1.53	32.58 ± 1.30	0.28	0.78

## Discussion

Prolonged hours of desk work are a commonly encountered occupational hazard at workplaces and a way of life for an increasing number of people in non-work contexts (Cohen 2015). The number of patients with secondary muscle spasms after stroke and other central nervous system diseases is on the rise (Phadke et al. 2019). These conditions can increase muscle tone in the splenius muscles, leading to cervical discomfort and pain. Intramuscular injection of BTX-A is a common and effective treatment for splenius muscle dystonia (Supiot et al. 2018). However, the optimal anatomical location of the injection has not been defined. Therefore, based on the distribution relationship between intramuscular nerves and muscle spindles in the splenius muscles, this study hypothesized that the CRHMSA in the INDR was the optimal target (Yu et al. 2023). Accurately localizing this target is clinically significant for guiding the administration of BTX-A injection and improving its efficiency in treating muscle dystonia.

The splenius capitis muscle is innervated by the posterior lateral branches of the C2 and C3 spinal nerves, while the nerves of the splenius cervicis muscle originate from the lateral branches of the posterior branches of the spinal nerves of the lower neck (Standring 2016). Besides reporting the same source of extramuscular nerves as described above, Kwon et al. reported that the C2 and C3 nerve branches innervating the splenius capitis muscle were densely distributed over approximately 50% of the muscle length. The nerves of the splenius cervicis muscle were mainly distributed over 30% to 70% of the muscle length (Kwon et al. 2020). In this study, the splenius capitis muscle was found to be innervated by the C1 nerve and the splenius cervicis muscle by the C3 nerve but not by the spinal nerves of the lower neck. The INDR of the splenius capitis and cervicis muscles ranged from 52.25 ± 0.77% to 62.80 ± 3.05% and 59.57 ± 1.83% to 63.96 ± 1.47%, respectively, which slightly differed from that reported by Kwon. Furthermore, this study also found that although there are special types of intramuscular nerve distribution in the splenius capitis muscle, the location of the INDR is consistent with the common type and does not affect the efficacy of BTX-A injection. However, there is no specific type of nerve distribution in the splenius cervicis muscle, perhaps due to the relatively small sample size in this study.

Regarding the target location for the intramuscular injection of BTX-A, studies using ultrasound guidance and autopsy have shown that the injection needle can be inserted into the splenius capitis muscle from the medial to the lateral side at the top of the posterior triangle of the neck at the C2 level (Ko et al. 2020). Another study used in vivo MRI scanning and combined ultrasound-guided and autopsy methods to identify the body surface landmark for injecting the splenius cervicis muscle. It found that the muscle could be assessed through the posterior triangle of the neck, between the C4 and C5 spinous process, from front to back, and horizontally through the levator scapulae muscle (Brumpt et al. 2021). These methods explain how BTX-A can be injected into the splenius muscles; however, they could not identify the precise location for optimum action. Kwon et al. reported that the INDR of the splenius capitis muscle, located at 50% of the muscle length, corresponds to the C5 vertebral body, while the INDR of the splenius cervicis muscle, located at 30–70% of the muscle length, corresponds to the T3 to C5 vertebral body. However, their study did not determine the optimal injection site and depth to accurately define the surface location.

BTX-A should maximize intrafusal muscle block and minimize extrafusal muscle block (Phadke 2013). When the upper motor neurons are affected, α and γ neurons are suppressed in the spinal cord, accompanied by hyperstretch reflex and associated muscle spasm. When gamma motor neuron activity increases, muscle spindle sensitivity increases, intensifying the activity of alpha motor neurons innervating the same muscle, resulting in increased muscle tension. Based on the theory that the muscle spindle abundance in INDR is unevenly distributed within the muscle, this study hypothesized that the area with the highest muscle spindle abundance in INDR is the optimal target for treatment. The results showed that the highest muscle spindle abundance in the two splenius muscles was in the middle of the INDR, with values slightly higher than those reported by Banks study (Banks 2006). This difference may be because the sampling part of the muscle used in this study was from the INDR with high muscle spindle abundance rather than from the average value of the whole muscle that was obtained in Bank's study.

In conclusion, this study found that CRHMSA of the splenius muscles is the optimal target for the injection of BTX-A to treat splenius muscle dystonia. Using the bony landmarks of the body surface, the relationship was established between the target and the body surface landmarks through the setting of longitudinal and horizontal reference lines. Labeling CRHMSA with barium sulfate, followed by spiral CT scanning and three-dimensional reconstruction, accurately determined the CRHMSA body surface percentage position and depth. This approach enhances the efficiency and efficacy of target localization. Adhering to these guidelines enables clinicians to use minimal doses, reduces the risk of adverse effects, such as antibody production and muscle fibrosis, and avoids the pain caused by multiple injections. Meanwhile, the guidelines can also be used for the efficient application of electromyography. However, this study has some limitations, including a relatively small sample size, lack of racial diversity in the samples studied, and no clinical validation of the anatomical findings. It is recommended that in the clinical application of BTX-A for the treatment of splenius muscle dystonia, BTX-A can be administered under ultrasound guidance, combined with the optimal target localization results of this study, but attention should be paid to the presence of blood vessels at the nerve entry sites of these two muscles.

## Data Availability

The data from this study will be made available upon reasonable request to the corresponding author.

## Abbreviations

BTX-A: Botulinum toxin A
CRHMSA: The center of the region of highest muscle spindle abundance
CT: Computed tomography
HE: Hematoxylin and eosin
INDR: Intramuscular nerve-dense region
L line: Longitudinal reference line
H line: Horizontal reference line
